# Voluntary wheel exercise training affects locomotor muscle, but not the diaphragm in the rat

**DOI:** 10.3389/fphys.2022.1003073

**Published:** 2022-10-26

**Authors:** Anna A. Borzykh, Dina K. Gaynullina, Anastasia A. Shvetsova, Oxana O. Kiryukhina, Ilya V. Kuzmin, Ekaterina K. Selivanova, Alexey M. Nesterenko, Olga L. Vinogradova, Olga S. Tarasova

**Affiliations:** ^1^ State Research Center of the Russian Federation, Institute of Biomedical Problems, Russian Academy of Sciences, Moscow, Russia; ^2^ Faculty of Biology, M.V. Lomonosov Moscow State University, Moscow, Russia; ^3^ Institute for Information Transmission Problems, Russian Academy of Sciences, Moscow, Russia; ^4^ Federal Center of Brain Research and Biotechnologies FMBA, Moscow, Russia; ^5^ Shemyakin-Ovchinnikov Institute of Bioorganic Chemistry of the Russian Academy of Sciences, Moscow, Russia

**Keywords:** voluntary exercise training, diaphragm, NADPH oxidase, SOD, OxPhos

## Abstract

**Introduction:** Functional tests and training regimens intensity-controlled by an individual are used in sport practice, clinical rehabilitation, and space medicine. The model of voluntary wheel running in rats can be used to explore molecular mechanisms of such training regimens in humans. Respiratory and locomotor muscles demonstrate diverse adaptations to treadmill exercise, but the effects of voluntary exercise training on these muscle types have not been compared yet. Therefore, this work aimed at the effects of voluntary ET on rat triceps brachii and diaphragm muscles with special attention to reactive oxygen species, which regulate muscle plasticity during exercise.

**Methods:** Male Wistar rats were distributed into exercise trained (ET) and sedentary (Sed) groups. ET group had free access to running wheels, running activity was continuously recorded and analyzed using the original hardware/software complex. After 8 weeks, muscle protein contents were studied using Western blotting.

**Results:** ET rats had increased heart ventricular weights but decreased visceral/epididymal fat weights and blood triglyceride level compared to Sed. The training did not change corticosterone, testosterone, and thyroid hormone levels, but decreased TBARS content in the blood. ET rats demonstrated higher contents of OXPHOS complexes in the triceps brachii muscle, but not in the diaphragm. The content of SOD2 increased, and the contents of NOX2 and SOD3 decreased in the triceps brachii muscle of ET rats, while there were no such changes in the diaphragm.

**Conclusion:** Voluntary wheel running in rats is intensive enough to govern specific adaptations of muscle fibers in locomotor, but not respiratory muscle.

## Introduction

Regular endurance exercise such as daily walking or moderate-speed running is beneficial for health, quality of life, and longevity (Leung et al., 2008; Ruegsegger and Booth, 2018; Burtscher and Burtscher, 2020). To achieve the best training result, exercise regimen should be adequate to the capabilities of the human body. Therefore, self-controlled functional tests and training regimens (such as 6-min walk distance test or tests/training sessions on non-motorized devices) are increasingly popular in sport practice, clinical rehabilitation, and space medicine (Janaudis-Ferreira et al., 2010; Yarmanova et al., 2015; Petersen et al., 2016; Wang et al., 2017; Penn et al., 2019).

Animal models provide mechanistic understanding of exercise physiological, biochemical, and molecular training effects in humans (Poole et al., 2020). Voluntary wheel training is an attractive model to study the impact of exercise on physiology and health outcomes in rodents (Poole et al., 2020). A great advantage of such training regimen is that it allows the animal to demonstrate its intrinsic motivation to exercise and, therefore, does not require aversive stimuli to motivate it to run. Notably, voluntary training in rats is not associated with an increase in blood corticosterone levels, in contrast to treadmill training (Ke et al., 2011; Sato et al., 2020). A voluntary exercise regimen seems to be aerobic since lactate accumulation in muscle interstitium should make the animal uncomfortable and force it to stop running. Such suggestion is supported by increases in the maximum oxygen consumption, citrate synthase activity and oxidative phosphorylation protein complexes in locomotor muscle observed after voluntary wheel training (Halseth et al., 1995; Toedebusch et al., 2016; Gaynullina et al., 2018). Importantly, a number of studies have reported beneficial effects of voluntary exercise training on rat nervous, muscular and cardiovascular systems as well as on the metabolic state (Przyklenk and Groom, 1985; Hägg et al., 2005; Hydock et al., 2007; Durrant et al., 2009; Gaynullina et al., 2018).

Endurance exercise increases the oxygen demand of working muscles and the whole body, which is met by the work of the respiratory muscles. Therefore, two functional types of skeletal muscle, locomotor, and respiratory ones, should increase their contractile activity during endurance exercise. The diaphragm, a principal mammalian inspiratory muscle, is rhythmically active throughout an organism’s life. The diaphragm has very high oxidative capacity and demonstrates a unique strategy of adaptation to endurance exercise training compared to locomotor muscles (Powers et al., 1997; Borzykh et al., 2020b). Following treadmill training, an increase of type I muscle fiber proportion and the decrease of muscle fiber cross-section area were observed in the diaphragm, which should facilitate the delivery and utilization of oxygen by diaphragm mitochondria (Green et al., 1989; Borzykh et al., 2012; Borzykh et al., 2017a). However, the oxidative capacity of the diaphragm is less susceptible to treadmill endurance training, in contrast to the locomotory muscle (Metzger and Fitts, 1986; Fregosi et al., 1987; Green et al., 1989; Powers et al., 1992; Uribe et al., 1992; Borzykh et al., 2012; Borzykh et al., 2017a). A vital function of the diaphragm necessitates the further search for new ways to support its aerobic performance. Therefore, the question arises about the comparison of voluntary exercise training effects on the oxidative capacity of respiratory and locomotory muscles in the rat. To our knowledge, this question had been poorly addressed in previous studies.

Reactive oxygen species (ROS) play an important role in the control of muscle tissue plasticity during exercise (Powers et al., 2011; Bouviere et al., 2021). Skeletal muscle contractile activity increases ROS production in muscle fibers (Powers et al., 2011; He et al., 2016; Powers, 2017; Thirupathi et al., 2021a). ROS are generated by the mitochondrial electron transport chain (predominantly by complexes I and III) or as products of enzymatic systems, among which NADPH oxidases (NOX) contribute significantly (Sakellariou et al., 2014; Knock, 2019; Tejero et al., 2019). NOX2 and NOX4 are dominant NOX isoforms in skeletal muscle, they produce superoxide anion (O_2_•−) and hydrogen peroxide (H_2_O_2_), respectively (Takac et al., 2011; Tejero et al., 2019). The balance of potentially more toxic O_2_•− and less toxic H_2_O_2_ is controlled by three classes of superoxide dismutase (SOD), which have distinct subcellular localization (Wang et al., 2018).

Notably, aerobic exercise can differently affect ROS-producing and antioxidant systems in an intensity and duration manner (Thirupathi et al., 2021b). Respiratory and locomotor muscles differ in regimens of contractile activity, which may create different conditions for ROS generation and inactivation. During an exhaustive treadmill running test, rat diaphragm was suggested to have higher antioxidant protection than locomotor muscles (Caillaud et al., 1999). However, no such comparison has been performed for voluntary wheel trained rats, to our knowledge. Furthermore, the effects of voluntary training on the expression of NOX and SOD isoforms in locomotor muscles and diaphragm have not been explored yet.

Therefore, this work aimed at the effects of voluntary exercise training on rat locomotor and respiratory muscles with special attention to the expression of NOX and SOD isoforms in muscle tissue. Training-induced alterations were compared in the diaphragm and triceps brachii muscle. This locomotor muscle is similar to the diaphragm in fiber type composition (Delp and Duan, 1999) and is substantially recruited during running in the wheel (Cohen and Gans, 1975). To confirm an increase of oxidative capacity of triceps brachii muscle following voluntary wheel training (Sexton, 1995; Toedebusch et al., 2016), we determined the contents of OXPHOS complexes in this muscle.

## Materials and methods

All experimental procedures used in this work were approved by the Biomedical Ethics Committee of the State Research Center of Russian Federation - Institute of Biomedical Problems, Russian Academy of Sciences (protocol N 574, approval date 12.04.2021), and conformed to the Guide for the Care and Use of Laboratory Animals published by the US National Institutes of Health (Eighth edition, 2011).

### Animals

Male Wistar rats were obtained from the vivarium of the State Research Center of Russian Federation—Institute of Biomedical Problems, Russian Academy of Sciences at the age of 2 months (body weight 250 ± 55 g, *n* = 24) and then housed in the animal unit of the Faculty of Basic Medicine, M.V. Lomonosov Moscow State University. The animals were maintained at the temperature 21–23°С on 12/12-h light/dark cycle (lighting on at 8:00 a.m., lighting off at 8:00 p.m.) and fed with normal rodent chow (Laboratorkorm, Russia) *ad libitum*.

### Voluntary wheel running

Exercise training of rats was carried out by voluntary wheel running. Rats (*n* = 24) were randomly assigned in pairs in 12 experimental setups. Rats were taken into the experiment by two per day, so that one experimental setup was inhabited each day.

The experimental setup was a cage (580 mm × 375 mm × 200 mm) with a running wheel (diameter 320 mm, the width of the running surface 100 mm) mounted on one side of the cage cover. Each wheel was equipped with two diametrically located magnets and a hall-effect sensor that was wired to an electronic counter (see our YouTube video https://youtu.be/OO7PnywRuvI). Each half-revolution of the wheel was recorded at the moment when the magnet passed the detector. Using the original software, the number of wheel half-revolutions was continuously recorded in intervals of 5 s and then the average running distance was calculated for each day of the training cycle (Borzykh et al., 2017b; 2020a). The source codes, mechanical drawings, and electronic circuits for our installation for animal spontaneous activity monitoring have been deposited in a public Github repository: https://github.com/comcon1/ASPAM. The codes for processing of spontaneous physical activity data generated by ASPAM can be downloaded from https://github.com/comcon1/ASPAM_processing.

After a week of rat adaptation to the experimental setup, the cage was separated by a mesh septum; as a result, the running wheel was constantly accessible only for one of the rats (from the exercise trained (ET) group) and constantly inaccessible for the second rat (sedentary (Sed) group). Throughout the experiment, the rats were able to interact through the holes in the septum, which eliminated the social isolation that could adversely affect the cardiovascular system (Sharp et al., 2002; Maslova et al., 2003). The exercise training period lasted for 8 weeks. During the experiment, the animals were weighed weekly. In post-training experiments, 23 rats (12 Sed and 11 ET) were used. One rat from the ET group was excluded due to a toe defect on the forelimb, it was paired in a setup with 12th rat from the sedentary group (in cage compartment without the wheel).

### Blood and tissue sampling

The running wheels were removed from the setups 20 h before the collection of blood and tissue samples. Each experimental day, two rats from the same setup were anesthetized with CO_2_ and killed by decapitation; for all animals euthanasia was performed at the same time of day (between 9:00 and 9:30 a.m.). Trunk blood samples were collected during decapitation. Blood samples were kept for 20 min at room temperature followed by 40 min at + 4°C, after which they were centrifuged (4,300 g for 15 min), serum was collected and stored at −20°C.

The long head of the triceps brachii muscle, right and left (together with the septum) heart ventricles, adrenal glands, epididymal and visceral fat were dissected and weighed. The muscle tissue samples were taken from the inner (red) part of the long head of the triceps brachii muscle and the costal part of the diaphragm. Muscle samples were quickly frozen in liquid nitrogen and stored at −70°C pending further Western Blotting experiments.

### Hormonal and biochemical analyses of blood serum

The hormone concentrations were determined using commercially available ELISA kits: testosterone, total thyroxine, and free triiodothyronine kits from Immunotek (Moscow, Russia) and rodent corticosterone kit from IDS (United Kingdom). Biochemical parameters were measured in an automatic analyzer (A-25 Biosystems, Spain). The concentrations of total cholesterol and triglycerides were determined using reagents from Hospitex Diagnostics (Russia). The concentrations of high-density lipoprotein (HDL) cholesterol and low-density lipoprotein (LDL) cholesterol were determined using reagents from Beijing Leadman Biochemistry (China). Thiobarbituric acid reactive substances (TBARS) levels were analyzed using the assay kit from Agat-Med (Russia).

### Western blotting

Seven rats were randomly selected from each (Sed and ET) group. Muscle tissue samples were homogenized in ice-cold RIPA buffer containing inhibitors of proteases and phosphatases (aprotinin 50 mg/ml, leupeptin 50 mg/ml, pepstatin 50 mg/ml, NaF 2.1 mg/ml, Na_3_VO_4_ 0.18 mg/ml). Homogenates were centrifuged at 14,000 g at 4°C for 5 min. Protein concentrations in supernatants were analyzed by bicinchoninic acid assays. Samples were mixed with 2x SDS sample buffer containing Tris 0.025 M (pH 6.8), SDS 2%, glycerol 20%, dithiothreitol 5.4%. An equal amount of total protein per lane (10 µg) was loaded to the 15-lane polyacrylamide gels, so that two compared groups of samples always ran in parallel (Supplementary Figures S1–S7). Proteins were separated by SDS-PAGE and transferred to nitrocellulose membrane (BioRad, United States) using Trans-Blot Turbo transfer system (BioRad, United States). The transfer was visualized with Ponceau S staining (Supplementary Figures S1–S7).

For further visualization of NOX2, NOX4, SOD1, SOD2, SOD3 parts of membranes were cut out at the appropriate level, in order to reduce the expenditure of antibodies (for detailed information see Supplementary Figures S4–S7). The membranes for further visualization of OXPHOS were processed uncut. All membranes were blocked with 5% nonfat milk (Applichem, Germany) in TBSt (20 mm Tris-HCl, pH 7.6; 150 mm NaCl, 0.1% Tween 20) and incubated overnight at +4°С with selected primary antibodies: a mixture of antibodies against oxidative phosphorylation complexes (CI-NDUFB8, CII-SDHB, CIII-UQCRC2, CIV-MTCO1, CV-ATP5A; OXPHOS, Abcam, 1:2000), NOX2 (Bioss Antibodies, 1:400), NOX4 (Abcam, 1:1,000), SOD1 (Sigma-Aldrich, 1:4,000), SOD2 (Enzo Life Science, 1:1,000) or SOD3 (Enzo Life Sciences, 1:1,000) in 5% milk in TBSt. The next morning the membranes were incubated with corresponding secondary antibodies (anti-mouse (Cell Signaling, 1:5,000 in 5% milk in TBSt) or anti-rabbit (Cell Signaling, 1:10,000 in 5% milk in TBSt)) for 1 h at room temperature and visualized with Super Signal West Dura Substrate (Thermo Scientific) using ChemiDoc (BioRad, United States).

Experiments were analyzed using ImageLab Software (BioRad, United States). To compare the protein content in two muscles of the sedentary group, the values were expressed as a percentage of the mean value for the triceps brachii muscle. When assessing the change in protein content after wheel training, the mean value for the muscle of the sedentary group was taken as 100%.

### Statistical data analysis

Statistical data analysis was performed using the GraphPad Prism 8.0 software. The normality of the data distribution was assessed using the Shapiro-Wilk test. For parameters with a normal distribution, data are presented as mean and S.D, the differences between groups were analyzed using the unpaired Student’s t-test (comparison of two groups), repeated measures one-way ANOVA (analysis of running activity indicators) or repeated measures two-way ANOVA (analysis of body weight dynamics in two groups); ANOVA tests were corrected for multiple comparisons. For samples with a distribution different from normal, the data are presented as the median and interquartile range; the differences between two groups were assessed using the Mann-Whitney test. Statistical significance was reached at *p* < 0.05, n is the sample size (number of animals).

## Results

### Characteristics of rat running activity

**FIGURE 1:**
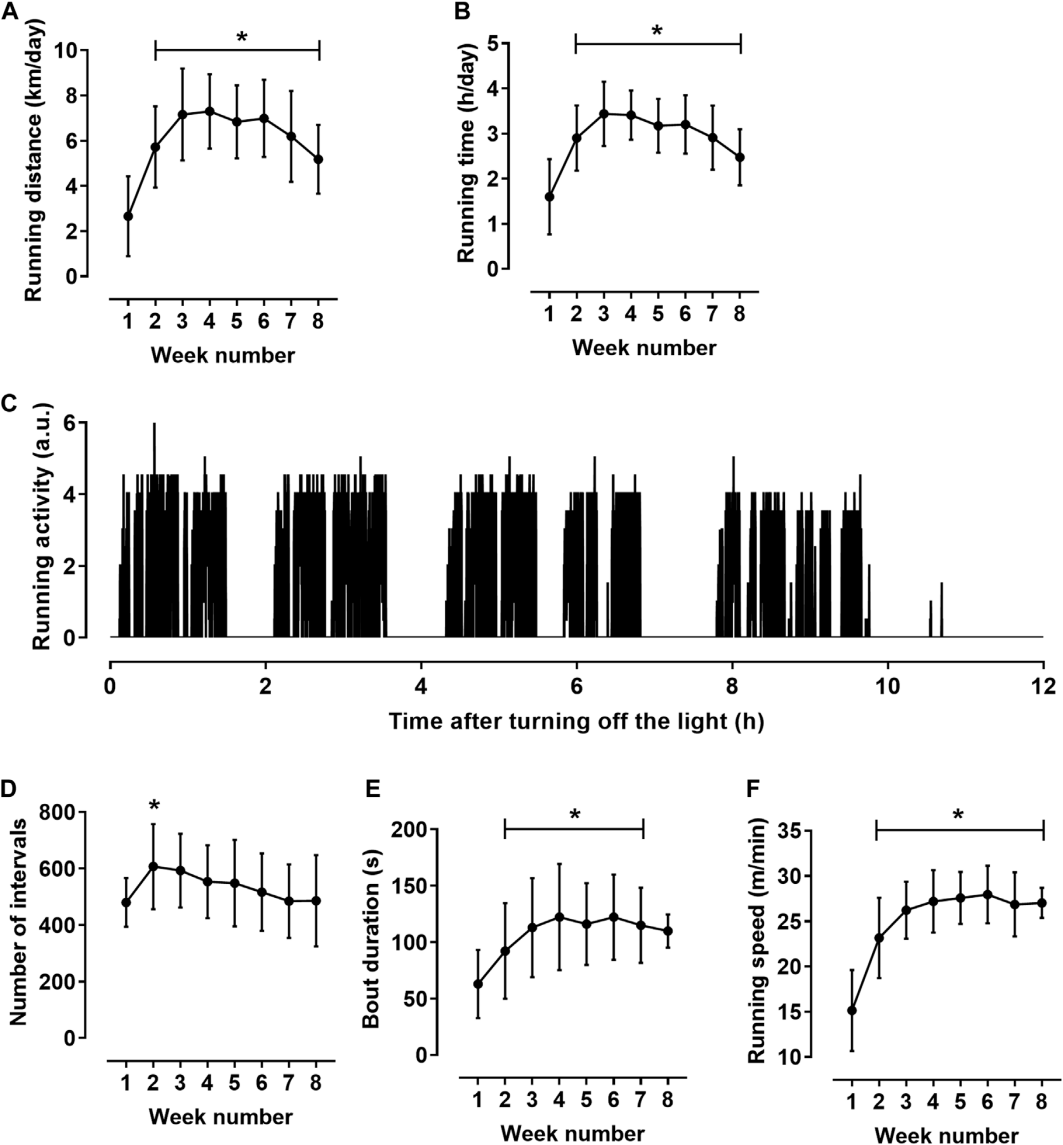
Characteristics of rat running activity during the 12-h dark period (*n* = 11). **(A,B)** daily running distance **(A)** and running time **(B)**. **(C)** Pattern of running activity of one rat at the seventh week of the training cycle. The initial value on the horizontal axis corresponds to the lighting off (at 8:00 p.m.); vertical axis shows the number of wheel half-revolutions in 5-s time intervals. **(D–F)** indicators calculated for individual bouts: numbers of bouts **(D)**, mean bout duration **(E)** and mean running speed in bouts **(F)** during the 8-week training cycle. *p < 0.05 compared to the first training week (Repeated measures one-way ANOVA with Dunnett’s multiple comparisons test).

The total run distance for 8 weeks was 340 ± 23 km. Rats exhibited their wheel-running activity predominantly during the dark period. On average, during most of the training cycle, the day running distance was about 1.5% of the night distance. Therefore, we performed detailed analysis of running activity during 12-h dark period. Daily run distance increased during the first 2 weeks of the exercise training, then plateaued and decreased slightly at the end of the training cycle **(** Figure 1A). Daily running time demonstrated similar dynamics (Figure 1B).

The activity was not evenly distributed in time but was observed on local time intervals lasting about an hour, which, in turn, were composed of shorter running bouts (Figure 1C). To characterize rat running activity in more detail, we calculated the values of the duration and speed of running in individual bouts. The bouts were defined as running intervals separated by at least 15-s intervals of rest (zero counts of wheel rotation in three consecutive 5-s intervals). Obtained values were averaged first for each 12-h dark period, and then weekly. The number of running bouts increased clearly from the first to the second week of training cycle and then gradually decreased (Figure 1D). Notably, a decrease in the number of bouts was accompanied by an increase in their duration and running speed (Figures 1E,F). Mean bout duration (Figure 1E) and mean running speed (Figure 1F) increased at the beginning of the training cycle, and then reached a plateau. From the third to the seventh week of the training cycle, bout duration was 117 ± 37 s, and the running speed was 27.2 ± 3.0 m/min. Notably, the coefficient of variation was least for running speed (5.3 ± 1.9%) compared to other indicators of running activity, such as distance (14.2 ± 7.9%), running time (12.1 ± 7.3%) and bout duration (12.1 ± 5.7%) (*p* < 0.05 for all comparisons, one-way ANOVA with Sidak’s multiple comparisons test).

### The effects of voluntary wheel running on organ weights and blood parameters

**FIGURE 2:**
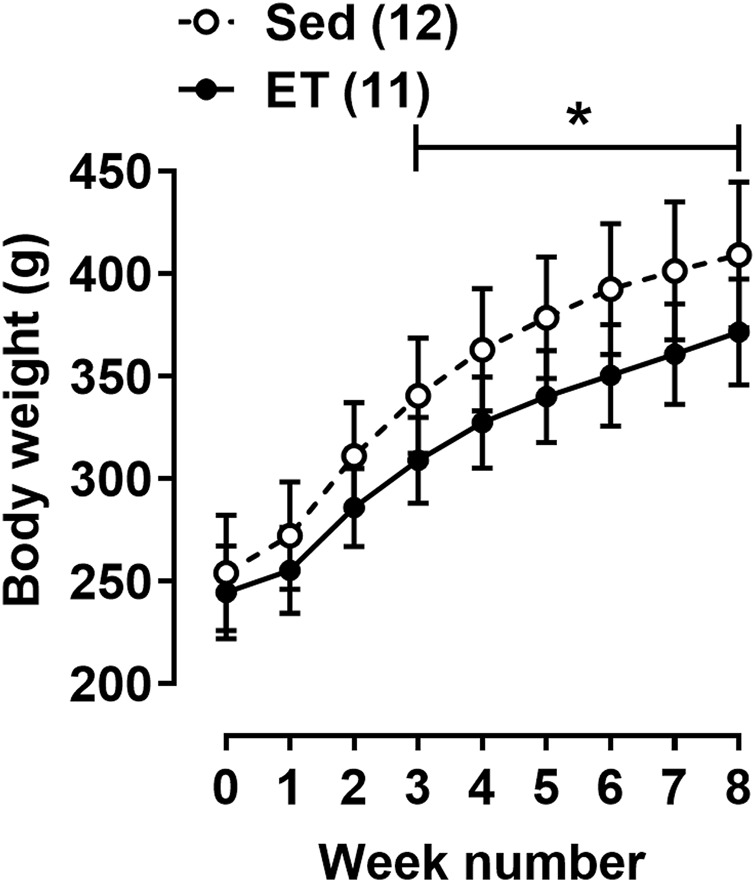
Body weight dynamics of rats during the experiment. Values at week “0” were obtained at the first day of the training cycle. Sed–sedentary group of rats; ET–exercise-trained group of rats; number in parentheses indicates the number of animals. *p < 0.05 compared to the sedentary group (two-way ANOVA with Sidak’s multiple comparisons test).

The body weight at the beginning of the training cycle did not differ between the two groups of rats (Figure 2). Starting from the third week of the training protocol till its end, the body weight of rats from ET group was about 10% less compared to the sedentary group (Figure 2). Voluntary wheel running did not change the weight of the triceps brachii muscle, but the weights of the right and left heart ventricles were 15% and 10% higher in ET rats compared to Sed group (Table 1). Notably, this type of exercise training seems to be not stressful for animals, since the weight of the adrenal glands, as well as the blood concentration of corticosterone, did not differ between the two groups of rats (Table 1). The blood levels of testosterone, thyroid hormones, cholesterol, HDL-cholesterol and LDL-cholesterol did not differ between ET and Sed groups (Table 1). At the same time, blood content of triglycerides was reduced by 22% in trained rats compared to sedentary ones, which correlated with a decrease in the relative weight of visceral and epididymal fat by 38% and 61%, respectively (Table 1). The content of TBARS in the blood serum of trained rats was reduced compared to sedentary ones (Table 1).

**TABLE 1:** Body weight (BW), relative organ weights and blood serum levels of hormones, substrates and thiobarbituric acid reactive substances (TBARS) in sedentary (Sed) and voluntary exercise-trained (ET) rats.

Parameters	Sed (n = 12)	ET (n = 11)
Body weight (g)	425 (389–448)	389 (367–404)^a^
Triceps brachii muscle long head (mg/100 g BW)	335 (316–353)	349 (332–375)
Right ventricle (mg/100 g BW)	52 (49–58)	61 (55–69)^a^
Left ventricle with septum (mg/100 g BW)	194 (188–216)	217 (208–222)^a^
Adrenal glands (both) (mg/100 g BW)	16.3 (14.4–17.3)	16.6 (15.7–18.5)
Visceral fat (g/100 g BW)	1.2 (1.0–1.4)	0.4 (0.3–0.4)^a^
Epididymal fat (g/100 g BW)	1.2 (1.0–1.5)	0.7 (0.7–0.8)^a^
Corticosterone (ng/ml)	21.5 (15.9–29.9)	20.0 (14.2–26.3)
Total thyroxine (nmol/L)	41.7 (31.7–47.1)	37.0 (30.8–45.2)
Free triiodothyronine (pmol/L)	4.3 (3.3–5.6)	4.7 (4.1–5.7)
Testosterone (nmol/L)	12.6 (5.3–21.8)	10.3 (4.2–16.4)
Cholesterol (mmol/L)	1.38 (1.22–1.77)	1.26 (1.22–1.37)
Triglycerides (mmol/L)	0.91 (0.78–1.24)	0.76 (0.70–0.90)^a^
HDL-Cholesterol (mmol/L)	1.0 (0.92–1.23)	0.88 (0.84–1.01)
LDL-Cholesterol (mmol/L)	0.19 (0.17–0.23)	0.19 (0.17–0.20)
TBARS (μmol/L)	43.8 (36.7–56.4)	33.8 (31.5–38.6)^a^

^a^ *p* < 0.05 compared to sedentary group (Mann-Whitney test). Number in parentheses indicates number of animals.

### The effects of voluntary wheel running on the contents of oxidative phosphorylation complexes in diaphragm and triceps brachii muscles

**FIGURE 3:**
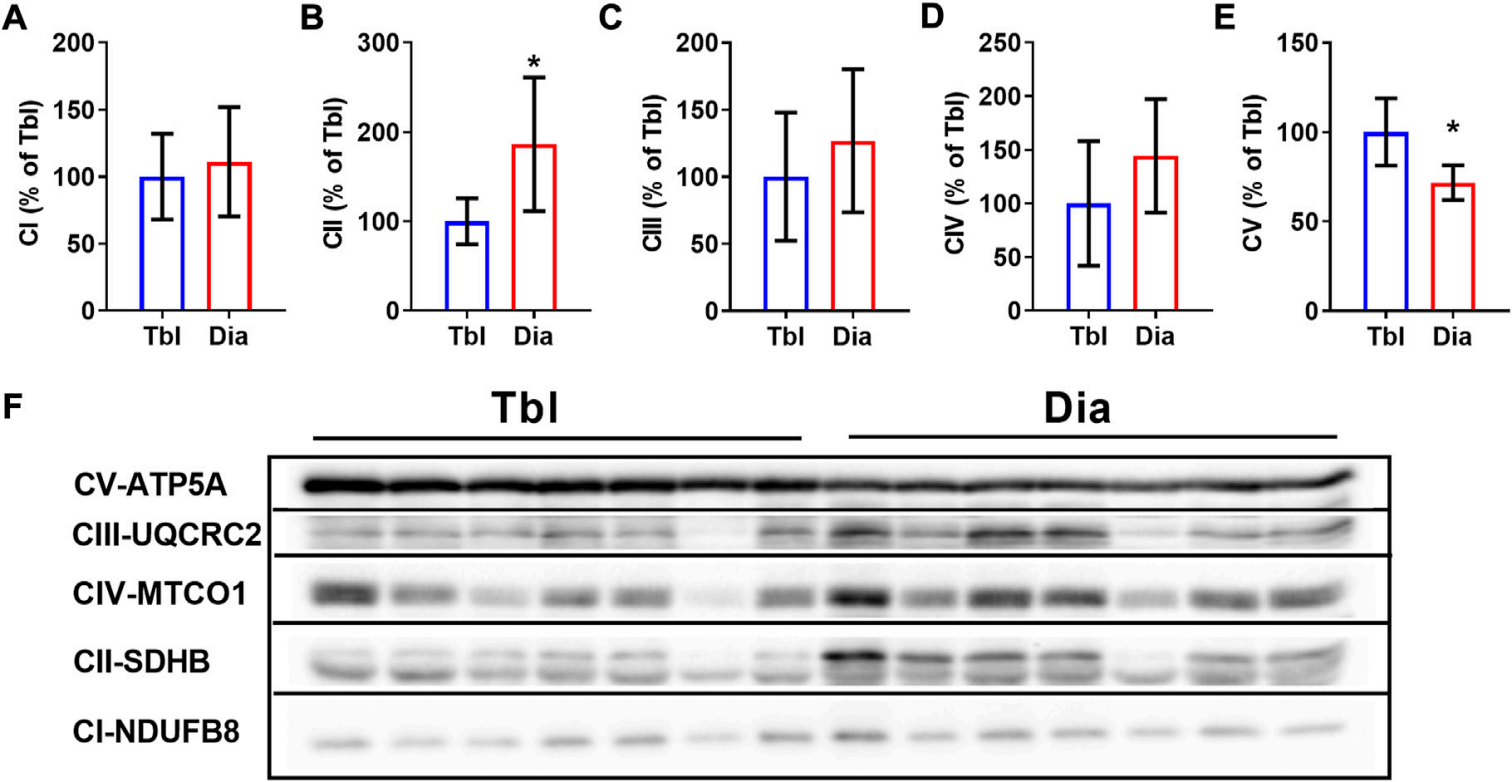
Rat triceps brachii muscle and diaphragm muscle differ in the contents of oxidative phosphorylation complexes. **(A–E)** The relative contents of CI (A), CII (B), CIII (C), CIV (D) and CV (E) oxidative phosphorylation complexes in triceps brachii muscle long head (Tbl) and diaphragm muscle (Dia) of sedentary rats (*n* = 7). **(F)** Representative Western Blot images of triceps brachii and diaphragm muscles oxidative phosphorylation complexes in sedentary rats. Images of CI-CV complexes were taken at different exposure times. *p < 0.05 compared to Tbl (unpaired Student’s t-test).

In sedentary rats, the content of the oxidative phosphorylation complex CII in the diaphragm was 86% higher than in the triceps brachii muscle (Figures 3B,F), the contents of complexes CI, CIII, and CIV did not differ between two muscles (Figures 3A,C,D,F). The content of the CV complex was somewhat (by 28%) lower in the diaphragm compared to the triceps brachii muscle (Figures 3E,F).

**FIGURE 4:**
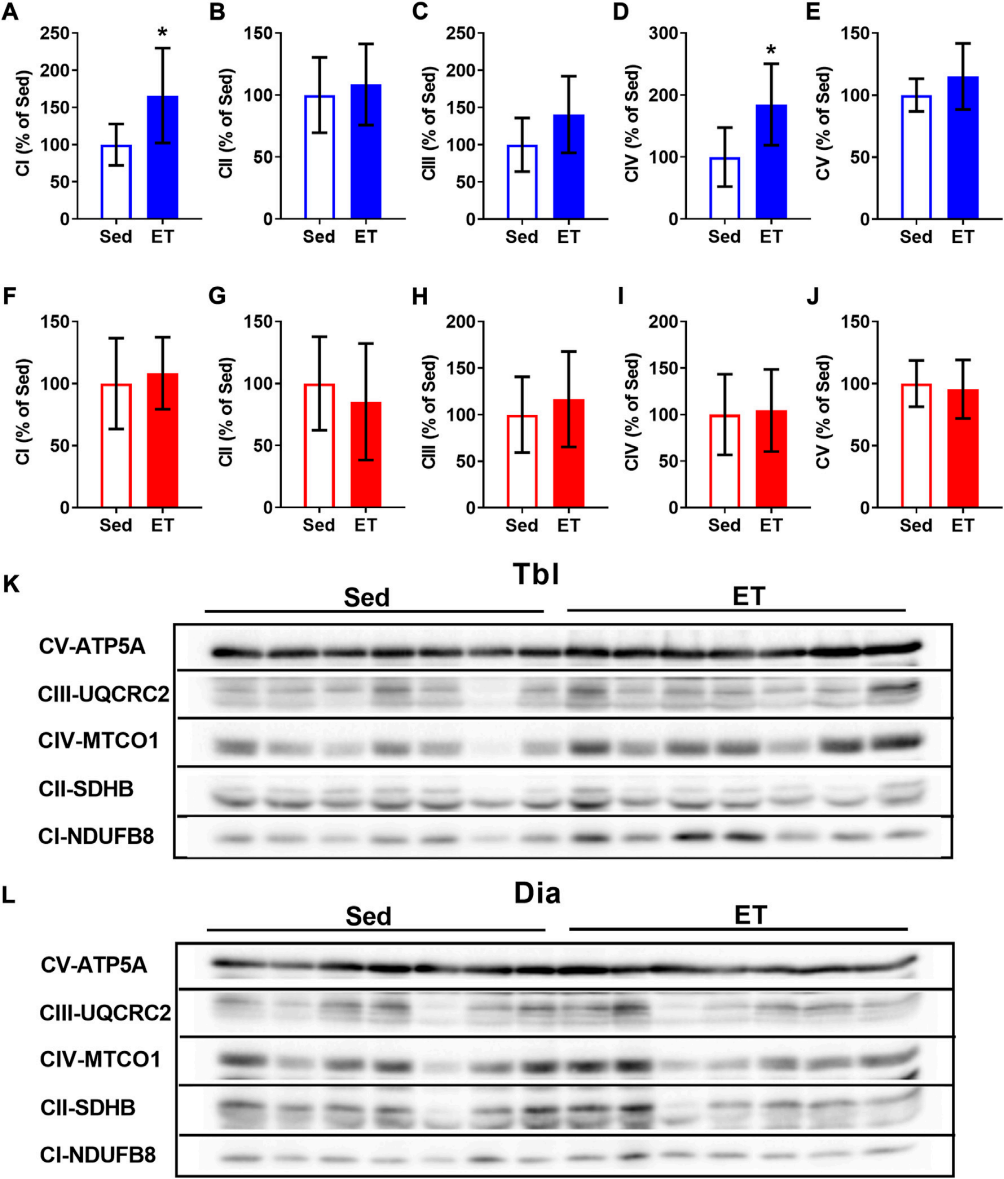
Voluntary exercise training increased the contents of oxidative phosphorylation complexes in long head of the triceps brachii muscle (Tbl) but not in diaphragm muscle (Dia). The relative contents of CI **(A,F)**, CII **(B,G)**, CIII **(C,H)**, CIV **(D,I)** and CV **(E,J)** oxidative phosphorylation complexes in Tbl **(A–E)** and Dia **(F–J)** of sedentary rats (Sed, *n* = 7) and voluntary exercise-trained (ET, *n* = 7) rats. Representative Western Blot images of oxidative phosphorylation complexes in Tbl **(K)** and Dia **(L)** in sedentary and voluntary exercise-trained rats. Images of CI-CV complexes were taken at different exposure times. *p < 0.05 compared to sedentary group (unpaired Student’s t-test).

In the triceps brachii muscle, ET increased the contents of CI and CIV oxidative phosphorylation complexes by 66% and 85% respectively (Figures 4A,D,K), while the contents of other oxidative phosphorylation complexes were not affected by ET (Figures 4B,C,E,K). In the diaphragm, the contents of oxidative phosphorylation complexes did not differ between sedentary and ET rats (Figures 4F–J,L). Thus, voluntary wheel running led to an increase in the oxidative potential of the forelimb muscle, but not the diaphragm.

### The effects of voluntary wheel running on the contents of prooxidant and antioxidant enzymes in diaphragm and triceps brachii muscles

**FIGURE 5:**
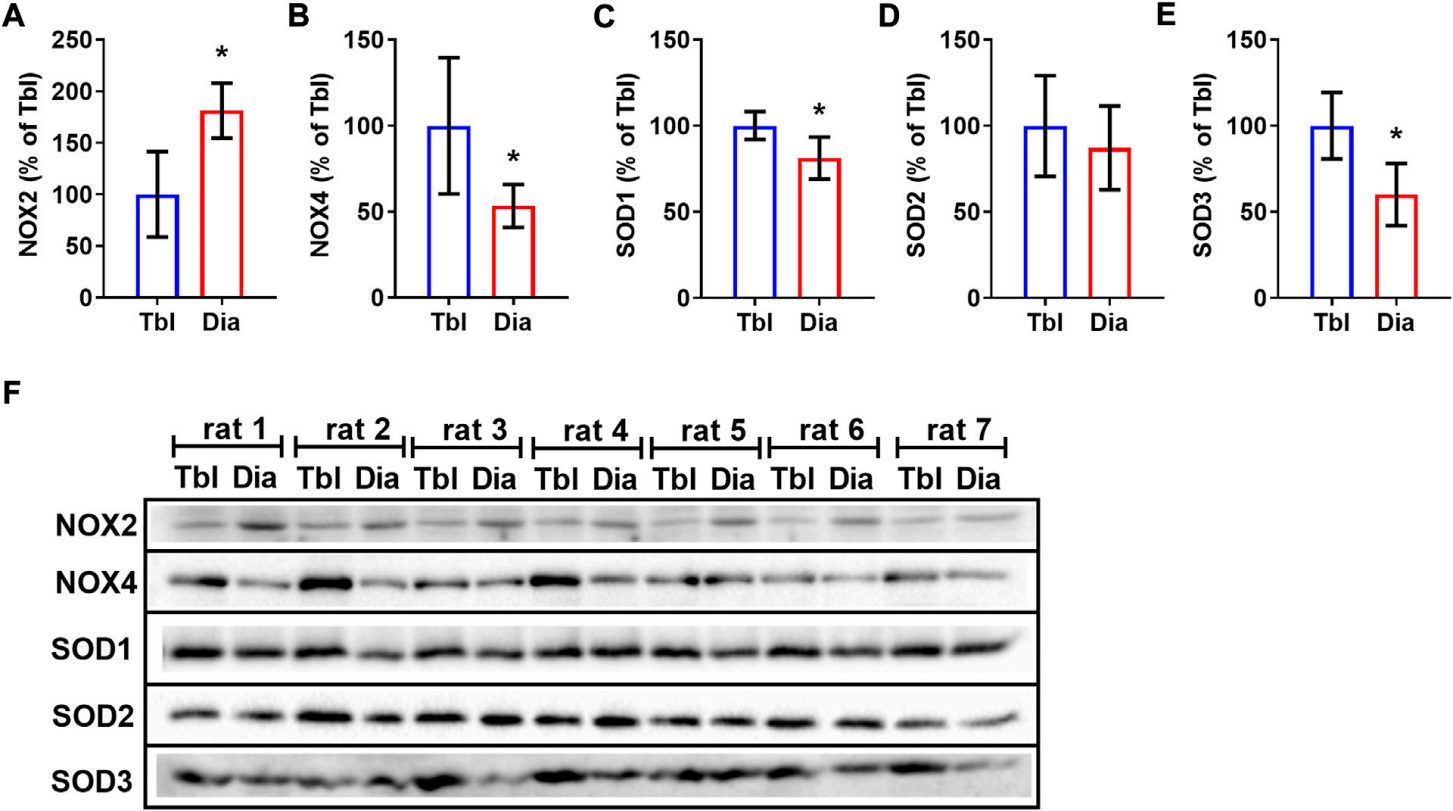
Rat triceps brachii muscle and diaphragm muscle differ in the contents of NOX and SOD isoforms. The relative contents of NOX2 **(A)**, NOX4 **(B)**, SOD1 **(C)**, SOD2 **(D)**, SOD3 **(E)** in triceps brachii muscle long head (Tbl, *n* = 7) and diaphragm muscle (Dia, *n* = 7) of sedentary rats **(A–E)**. Representative Western Blot images of NOXs and SODs isoform content in triceps brachii muscle and diaphragm muscle in sedentary rats **(F)**. *p < 0.05 compared to Tbl (unpaired Student’s t-test).

Sedentary rats had 81% higher levels of NOX2 in the diaphragm compared to triceps brachii muscle (Figures 5A,F) and, conversely, 47% lower levels of NOX4 (Figures 5B,F). Cytosolic (SOD1) and extracellular (SOD3) superoxide dismutase isoform contents were lower in the diaphragm compared to the triceps brachii muscle by 19% and 40% respectively (Figures 5C,E,F), while the mitochondrial SOD2 isoform (Figures 5D,F) did not differ between studied muscles.

**FIGURE 6:**
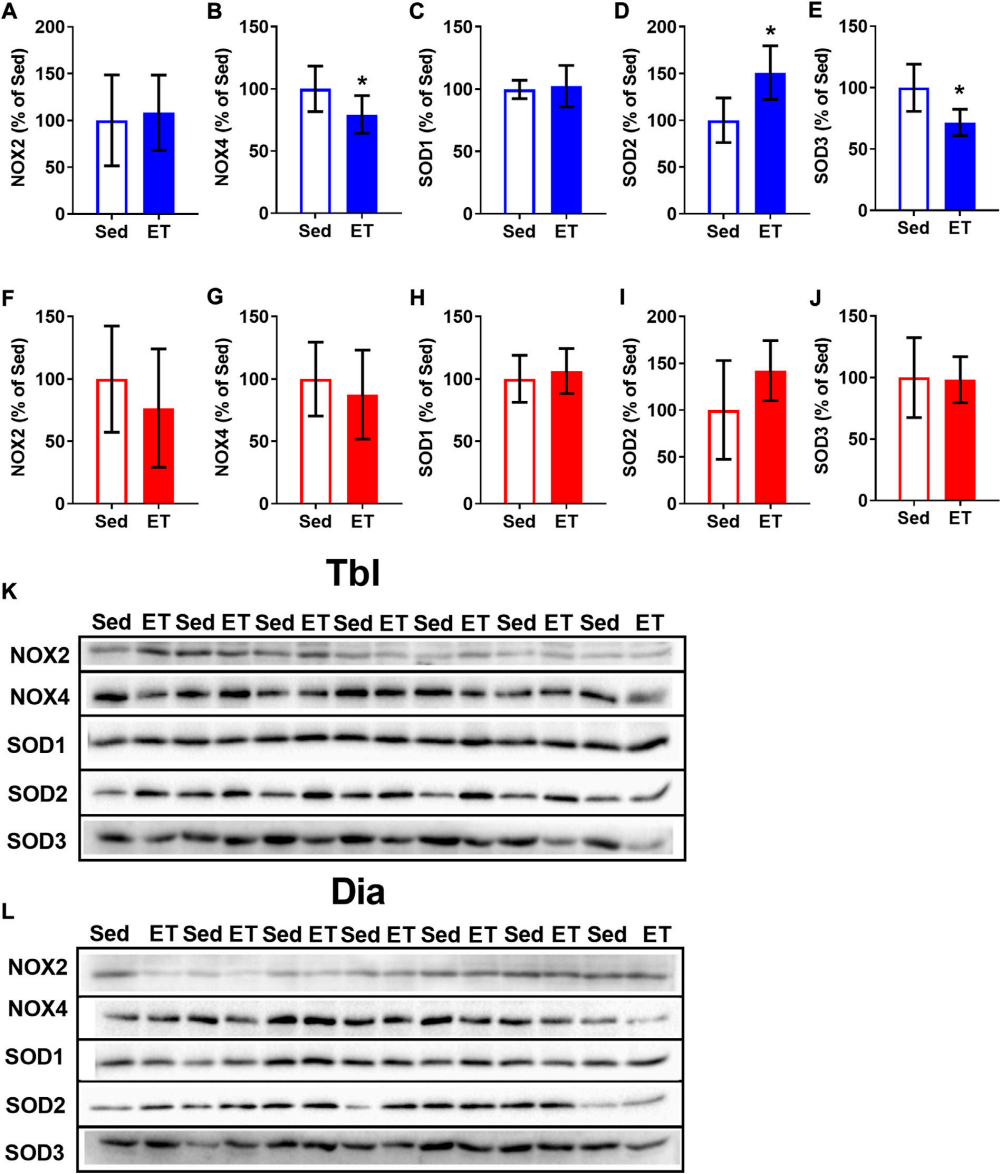
Voluntary exercise training changed the contents of NOX and SOD isoforms in long head of the triceps brachii muscle (Tbl) but not in diaphragm muscle (Dia). The relative contents of NOX2 **(A,F)**, NOX4 **(B,G)**, SOD1 **(C,H)**, SOD2 **(D,I)** and SOD3 **(E,J)** in Tbl **(A–E)** and Dia **(F–J)** of sedentary rats (Sed, *n* = 7) and voluntary exercise-trained (ET, *n* = 7) rats. Representative Western Blot images of NOX and SOD isoforms in Tbl **(K)** and Dia **(L)** of sedentary and voluntary exercise-trained rats. *p < 0.05 compared to sedentary group (unpaired Student’s t-test).

Voluntary ET did not alter the content of NOX2 in the triceps brachii muscle (Figures 6A,K), but reduced by 21% the content of NOX4 (Figures 6B,K). The effects of ET on SOD expression were also isoform-specific: SOD1 expression did not change (Figures 6C,K), SOD2 increased by 51% (Figures 6D,K), and SOD3 decreased by 28% (Figures 6E,K) as a result of voluntary wheel running. Along with that, ET did not change the contents of NOX and SOD isoforms in the diaphragm (Figures 6F–J,L).

## Discussion

Here we report a novel finding that voluntary wheel training exerts non-uniform effects on triceps brachii and diaphragm muscles in the rat. Triceps brachii muscle demonstrated an increase of oxidative capacity and substantial alterations in contents of ROS synthesis/degradation enzymes, while no such changes were apparent in the diaphragm.

### Characteristics of rat voluntary wheel running activity

Starting from the third week of the training cycle, our rats demonstrated a daily run distance in the range from 5 to 9 km. Based on the previously reported classification of rats by their voluntary running distance (Rodnick et al., 1989; Sexton, 1995; Poole et al., 2020) we can infer the medium level of voluntary running activity of our rats. Daily running distance, running time and the bout duration slightly decreased to the end of the training cycle, when the rats became about 18-week-old. Similar decrease of voluntary running activity in young male rats with increasing their age to 18 weeks was reported by Mondon and others (Mondon et al., 1985).

Wheel running activity occurred during the dark period and consisted of short bouts, similar to the results of previous studies (Mondon et al., 1985; Rodnick et al., 1989; Borzykh et al., 2017b; Poole et al., 2020). At the beginning of the training cycle, the increase in the total running distance was associated with an increase in the number of bouts. Afterwards, the number of bouts decreased, but their duration and speed of running increased. Probably, these changes reflect the acquisition of running skills by rats. Bout duration and running speed first increased and plateaued from the third training week. Mean running speed demonstrated by our young male rats (∼27 m/min) was close to the values that can be calculated from the data on wheel revolutions per minute and wheel diameter reported in previous studies (Mondon et al., 1985; Eikelboom and Mills, 1988).

Notably, running speed was the least variable and, probably, most informative characteristic of rat’s running activity in comparison to run distance, run time and bout duration. According to our previous data, rats are able to develop the individual tactics of wheel running (Borzykh et al., 2017b). During the training cycle, the distribution of instantaneous speed values gradually narrows and acquires a maximum, which corresponds to the running speed most characteristic of this rat. Apparently, each rat demonstrates an individual “comfortable” running speed. Probably, the speed of voluntary wheel running may be an indicator of the functional state of the organism. For example, wheel running speed in heart failure rats is one and a half times lower than in healthy rats (Schultz et al., 2013).

### Voluntary wheel running induces gross adaptations typical for endurance exercise training

The body weight of voluntary trained rats was lower compared to that in sedentary control, in part due to the decrease of fat weight, which is typical for endurance training (Lehnig and Stanford, 2018). Such shortage of adipose tissue correlated with the reduction of blood content of triglycerides pointing to their elevated utilization in physically active rats. Therefore, voluntary wheel training can be beneficial for lipid metabolism, preventing the development of insulin resistance and obesity (Reaven and Reaven, 1981; Matiello et al., 2010). Moreover, moderate heart hypertrophy was observed in voluntary trained rats, similar to observed earlier in rats with high voluntary running activity (Rodnick et al., 1989; Sexton, 1995). Heart hypertrophy is also a typical effect of endurance exercise training.

Importantly, no signs of chronic stress were observed in ET rats, as seen from unchanged adrenal weight and blood corticosterone level compared to those in sedentary rats. The hypothalamic-pituitary-thyroid axis was also not altered in voluntary trained rats, similar to earlier reported data (Gaynullina et al., 2018). Therefore, voluntary wheel running ensures the adaptation of the rat organism to chronic aerobic exercise in non-stressing conditions.

### Voluntary wheel training increases oxidative potential in locomotor, but not respiratory muscle

The oxidative capacity of the diaphragm is higher than that of most locomotor muscles (Delp and Duan, 1996; Borzykh et al., 2020b). Accordingly, we observed higher content of oxidative phosphorylation complex II in the diaphragm compared to the triceps brachii muscle. Notably, in a previous study we observed higher content of succinate dehydrogenase, a key component of complex II, in rat diaphragm compared to locomotor gastrocnemius muscle (Borzykh et al., 2017a). The content of ATP synthase subunit α, a marker of oxidative phosphorylation complex V, in the diaphragm was somewhat lower than in the triceps brachii muscle. It should be noted, however, that ATP synthase activity does not limit mitochondrial respiration in rat diaphragm (Callahan et al., 2001).

Movement analysis and electromyography in rats demonstrated the substantial activation of triceps brachii muscle during walking and running in the wheel (Cohen and Gans, 1975). Therefore, the oxidative capacity of this muscle increases following voluntary wheel training, as seen from the elevations of citrate synthase activity (Sexton, 1995; Toedebusch et al., 2016) and the contents of oxidative phosphorylation complexes (Toedebusch et al., 2016). In our study, the contents of oxidative phosphorylation complexes I and IV were higher in triceps brachii muscle of voluntary trained rats compared to sedentary control. However, no such differences were seen in the diaphragm muscle. Our results are supported by an earlier published study that showed less pronounced metabolic changes in the diaphragm compared to hindlimb muscles of voluntary exercise-trained rats (Halseth et al., 1995). Therefore, oxidative capacity of permanently active diaphragm muscle hardly demonstrates an additional rise during voluntary exercise training.

Qualitatively similar results had been observed in rats trained on a motor-driven treadmill (Metzger and Fitts, 1986; Green et al., 1989; Okumoto et al., 1996; Borzykh et al., 2012, 2017a). Due to the different biomechanical characteristics of locomotion, wheel running, and treadmill running are hardly comparable in terms of load intensity and volume. However, it can be assumed that treadmill running is not less energy-demanding than wheel running, since treadmill speed usually exceeds 30 m/min, and besides, the incline of treadmill lane further increases the load. Therefore, diaphragm muscle is less susceptible to changes in its oxidative capacity during different regimens of endurance training, due to its intrinsically high oxidative capacity associated with permanent contractile activity.

### Voluntary wheel training induces specific changes of ROS metabolism in locomotor, but not respiratory muscle

Adaptive alterations of muscle fibers during acute and chronic exercise are driven in part by self-generated ROS (Powers et al., 2011; Bouviere et al., 2021). Since ROS production differs in fast and slow muscle fibers (Loureiro et al., 2016), we compared the contents of key prooxidant and antioxidant enzymes in the diaphragm and triceps brachii muscle that are similar in fiber composition (Delp and Duan, 1996). Basal levels of NOX isoforms demonstrated non-uniform differences between the two studied muscles: the level of O_2_•− producing NOX2 was higher in the diaphragm, while the level of H_2_O_2_ producing NOX4 was higher in triceps brachii muscle. These data suggest a higher basal rate of O_2_•− production in the diaphragm. Surprisingly, the levels of expression of two of the three SOD isoforms were lower in the diaphragm compared to triceps brachii muscle, which is in contradiction with the data on higher antioxidant enzyme capacity of the diaphragm (Ragusa et al., 1996; Talarmin et al., 2017). It should be noted, however, that earlier reported data were obtained by comparing the diaphragm with hindlimb muscles that have different muscle fiber composition. In addition, antioxidant protection of the diaphragm may not be associated with SOD, but with other antioxidant enzymes.

Chronic endurance exercise can cause shifts in expression/activity of both prooxidant and antioxidant systems in skeletal muscle (Bouviere et al., 2021). In principle, endurance exercise is capable to increase SOD expression/activity in rat diaphragm but all the data we know relate to treadmill training (Lawler et al., 1994; Powers and Criswell, 1996; Oh-ishi et al., 1997). Of note, endurance exercise was shown to improve oxidative stress in the diaphragm in mouse model of Duchenne muscular dystrophy (Nocetti et al., 2021) and in patients with chronic obstructive pulmonary disease (Zhang et al., 2021). Comparative studies of the diaphragm and locomotor muscles in this aspect are limited to the effects of acute treadmill exhaustive running test, during which changes in antioxidant enzyme activities were less pronounced in the diaphragm compared to that in hindlimb muscles (Caillaud et al., 1999). For the first time, we showed that chronic voluntary exercise did not affect the contents of NOX and SOD isoforms in the rat diaphragm although obvious changes of these indicators were observed in the triceps brachii muscle. Presumably, voluntary training reduced the differences in NOX4 and SOD3 contents between two studied muscles, since inherently high contents of these proteins in triceps brachii muscle were reduced by training. Our data show that voluntary exercise training ensured the protection of locomotor muscle from oxidative stress, as seen by the decrease of NOX4 expression and, importantly, by the increase of SOD2 expression, a key beneficial effect of endurance exercise (Powers et al., 2011).

Notably, voluntary exercise training was accompanied by the decrease of TBARS content in rat blood. Although acute endurance exercise may increase blood TBARS content in volume-dependent manner (Vezzoli et al., 2016), the effect of chronic exercise training is rather the opposite, as physically active people have lower TBARS in the resting state (Djordjevic et al., 2012). Importantly, in our study the rats were deprived of exercising almost a day before blood sampling. Our results are consistent with lower basal TBARS levels in blood of rats after other training methods: moderate swimming protocol (Stanojevic et al., 2016) or treadmill running (Delwing-de Lima et al., 2018). Therefore, the systemic antioxidant protection of tissues can be a general beneficial effect of the voluntary exercise training.

Our present study has several limitations/unexplored topics that should be addressed in future studies, including the determination of NOX and SOD specific activities in muscle tissue as well as the levels of expression/activity of other antioxidant enzymes: glutathione peroxidase, catalase etc.

## Conclusion

For the first time, we performed a comprehensive analysis of voluntary wheel training effects on locomotor and respiratory muscles in rats. We report the novel findings that chronic voluntary wheel exercise causes less marked changes in oxidative capacity and prooxidant/antioxidant enzyme expression in the diaphragm compared to the triceps brachii muscle, which is matched to the diaphragm in muscle fiber type composition. Considering the characteristics of these two muscles and the training principle of physiological overload it can be concluded that voluntary running is demanding for the brachial muscle but not for the diaphragm. Therefore, in healthy young rats the diaphragm does not develop specific adaptations in response to spontaneous and intermittent exercise bouts that do not cause its physiological overload.

Importantly, the principle of rat voluntary training relates to human self-controlled intensity exercise protocols that are used in astronauts (Yarmanova et al., 2015; Petersen et al., 2016), stroke patients (Wang et al., 2017) or Parkinson’s disease patients (Penn et al., 2019), to assess and develop their aerobic performance. Therefore, the results of the present study may be considered in exploring the systemic effects of such training protocols and the development of training programs aimed at increasing aerobic performance of skeletal muscles.

## Data Availability

The raw data supporting the conclusions of this article will be made available by the authors, without undue reservation.
