# Supplementary material for: Voluntary wheel exercise training affects locomotor muscle, but not the diaphragm in the rat
Source: Front Physiol. 2022 Oct 26;13:1003073. doi: 10.3389/fphys.2022.1003073 (PMC9643685; doi:10.3389/fphys.2022.1003073)
Supplement: Supplementary file 1 [file DataSheet1.PDF]

Supplementary Material

Membrane 1 after Ponceau S staining

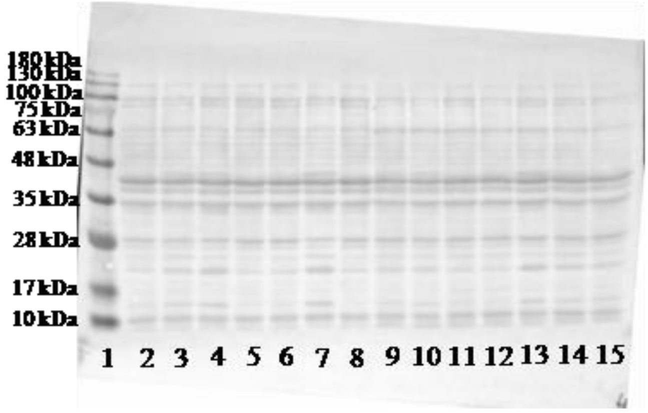

Lane 1: protein ladder (ab116027)

Lanes 2 – 8: **triceps brachii muscles of sedentary rats**

Lanes 9 – 15: **diaphragm muscles of sedentary rats**

Oxidative phosphorylation complexes (OXPHOS)	Super Signal West Dura Substrate (ECL) dilution in TBST - exposure time (s)	Original unprocessed images of membrane 1 → - band for analyses
CV-ATP5A (55kDa)	1:5 – 2 s	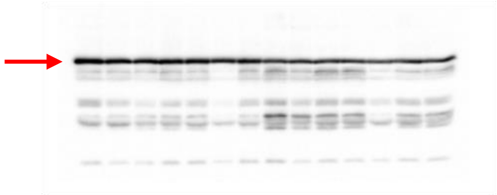
CIII-UQCRC2 (48 kDa)	1:5 – 7 s	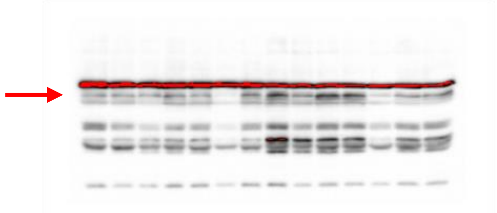
CIV-MTCO1 (40 kDa)	1:5 – 11 s	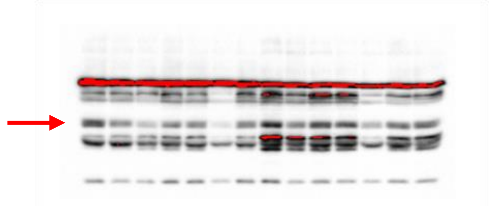
CII-SDHB (30 kDa)	1:5 – 5 s	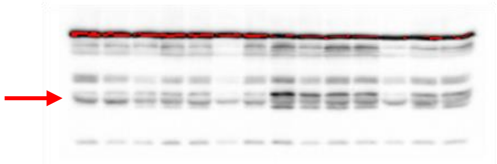
CI-NDUFB8 (20 kDa)	1:1 – 1 s	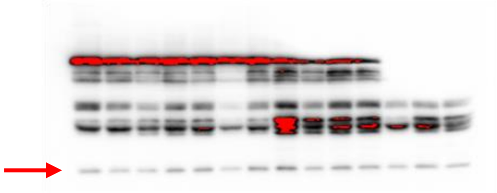

Supplementary FIGURE S1 Ponceau S staining and unprocessed images of membrane 1 stained with OXPHOS antibody cocktail in long head of triceps brachii muscle and diaphragm muscle of sedentary rats (n=7). Images of CI-CV complexes were taken at different exposure times.

Membrane 2 after Ponceau S staining

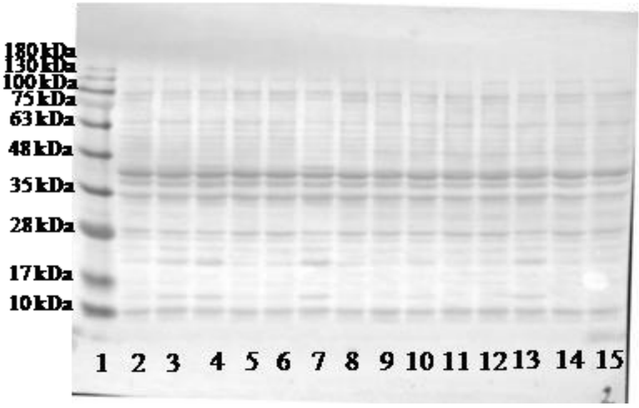

Lane 1: protein ladder (ab116027)
Lanes 2 – 8: **triceps brachii muscles of sedentary rats**

Lanes 9 – 15: **triceps brachii muscle of voluntary exercise-trained rats**

Oxidative phosphorylation complexes (OXPHOS)	Super Signal West Dura Substrate (ECL) dilution in TBST - exposure time (s)	Original unprocessed images of membrane 2 → - band for analyses
CV-ATP5A (55kDa)	1:5 – 3 s	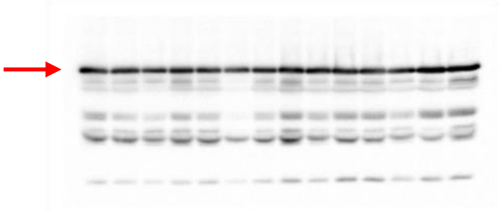
CIII-UQCRC2 (48 kDa)	1:5 – 8 s	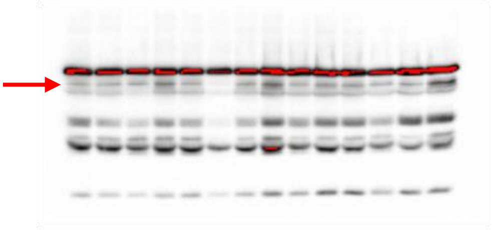
CIV-MTCO1 (40 kDa)	1:5 – 9 s	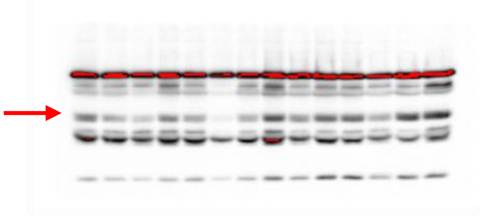
CII-SDHB (30 kDa)	1:5 – 5 s	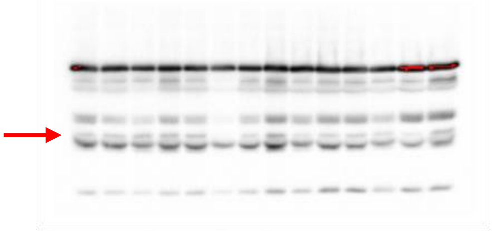
CI-NDUFB8 (20 kDa)	1:1 – 1 s	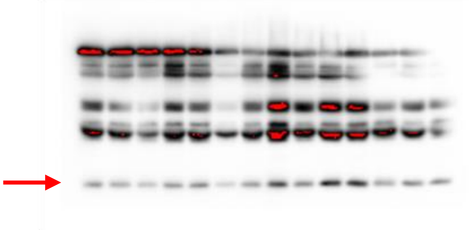

Supplementary FIGURE S2 Ponceau S staining and unprocessed images of membrane 2 stained with OXPHOS antibody cocktail in long head of triceps brachii muscle of sedentary (n=7) and voluntary exercise-trained (n=7) rats. Images of CI-CV complexes were taken at different exposure times.

Membrane 3 after Ponceau S staining

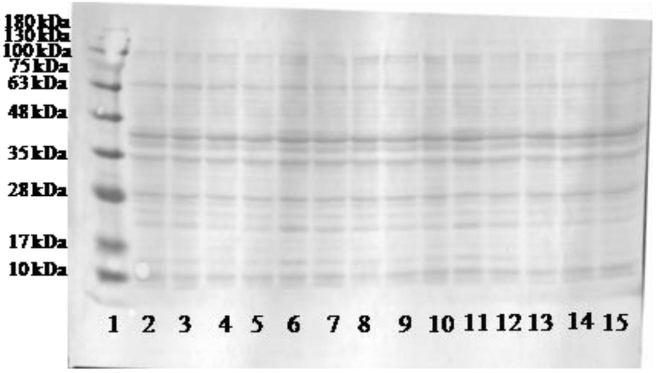

Lane 1: protein ladder (ab116027)
Lanes 2 – 8: **diaphragm muscles of sedentary rats**
Lanes 9 – 15– **diaphragm muscle of voluntary exercise-trained rats**

Oxidative phosphorylation complexes (OXPHOS)	Super Signal West Dura Substrate (ECL) dilution in TBST - exposure time (s)
CV-ATP5A (55kDa)	1:5 – 3 s
CIII-UQCRC2 (48 kDa)	1:5 – 8 s
CIV-MTCO1 (40 kDa)	1:5 – 9 s
CII-SDHB (30 kDa)	1:5 – 5 s
CI-NDUFB8 (20 kDa)	1:1 – 1 s

Original unprocessed images of membrane 3
→ - band for analyses

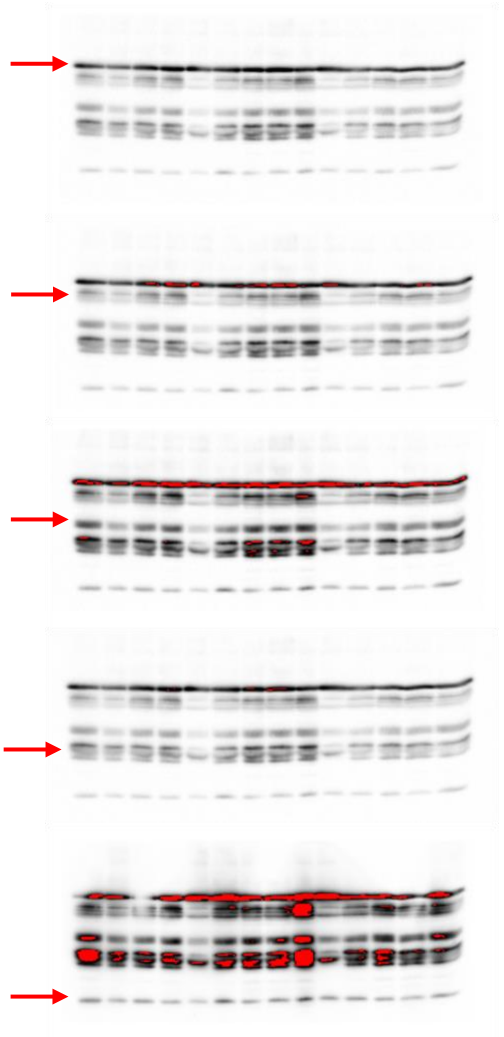

Supplementary FIGURE S3 Ponceau S staining and unprocessed images of membrane 3 stained with OXPHOS antibody cocktail in diaphragm muscle of sedentary (n=7) and voluntary exercise-trained (n=7) rats. Images of CI-CV complexes were taken at different exposure times.

Membrane 4 after Ponceau S staining

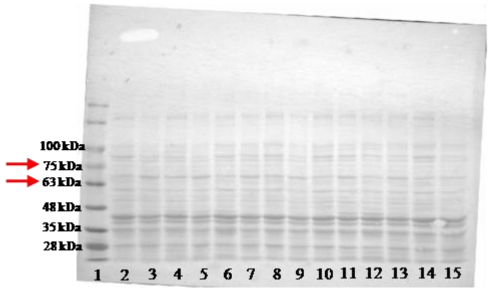

Lane 1: protein ladder (ab116027)
Even lanes: **triceps brachii muscles of sedentary rats**
Odd lanes: **diaphragm muscles of sedentary rats**
→ - the membrane was cut between 75 kDa (top) and 63 kDa (bottom) for further visualization of NOX2 (approx. 65 kDa).

Fragment of membrane 4 (75 kDa – 63 kDa) stained with anti-NOX2 antibodies

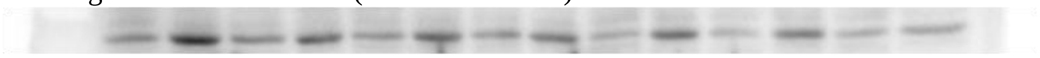

Membrane 5 after Ponceau S staining

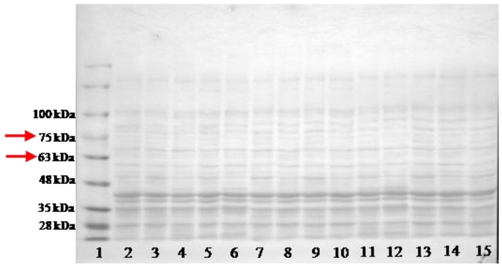

Lane 1: protein ladder (ab116027)
Even lanes: **triceps brachii muscles of sedentary rats**
Odd lanes: **triceps brachii muscles of voluntary exercise-trained rats**
→ - membrane was cut between 75 kDa (top) and 63 kDa (bottom) for further visualization of NOX2 (approx. 65 kDa).

Fragment of membrane 5 (75 kDa – 63 kDa) stained with anti-NOX2 antibodies

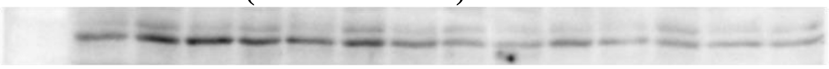

Membrane 6 after Ponceau S staining

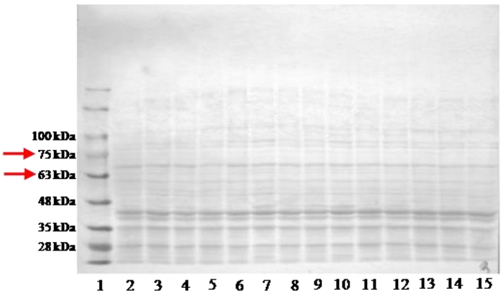

Lane 1: protein ladder (ab116027)
Even lanes – **diaphragm muscles of sedentary rats**
Odd lanes – **diaphragm muscles of voluntary exercise-trained rats**
→ - membrane was cut between 75 kDa (top) and 63 kDa (bottom) for further visualization of NOX2 (approx. 65 kDa).

Fragment of membrane 6 (75 kDa – 63 kDa) stained with anti-NOX2 antibodies

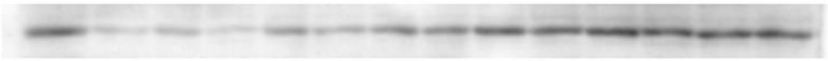

Supplementary FIGURE S4 Ponceau S staining and unprocessed images of membrane fragments stained with antibodies against NOX2. Membrane 4 – in long head of triceps brachii muscle and diaphragm muscle of sedentary rats (n=7); membrane 5 – in long head of triceps brachii muscle of sedentary (n=7) and voluntary exercise-trained (n=7) rats; membrane 6 – in diaphragm muscle of sedentary (n=7) and voluntary exercise-trained (n=7) rats. The fragment of membrane (at the level of the NOX2) was cut out to reduce the quantity of antibodies used.

Membrane 7 after Ponceau S staining

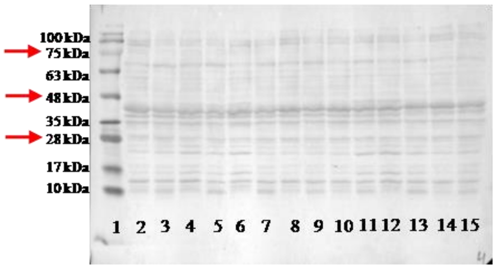

Lane 1: protein ladder (ab116027)
Even lanes: **triceps brachii muscles of sedentary rats**
Odd lanes: **diaphragm muscles of sedentary rats**
→ - membrane was cut into three parts:
between 75 and 48 kDa (top) for NOX4 (67 kDa);
between 48 and 28 kDa (middle) for SOD3 (35 – 40 kDa);
below 28 kDa (bottom) for SOD1 (18 kDa).

Top fragment of membrane 7 stained against NOX4

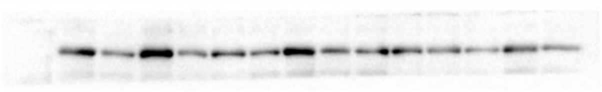

Middle fragment of membrane 7 stained against SOD3

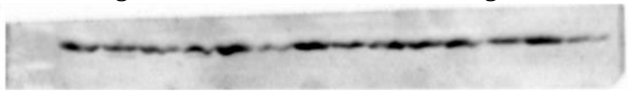

Bottom fragment of membrane 7 stained against SOD1

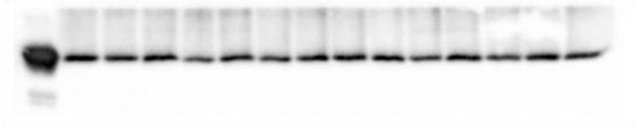

Membrane 8 after Ponceau S staining

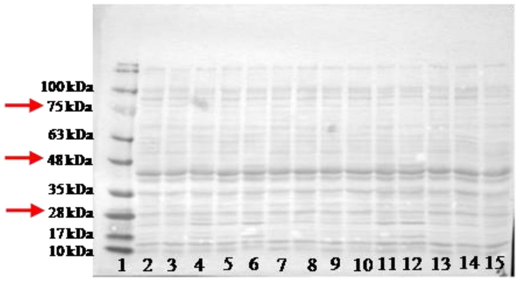

Lane 1: protein ladder (ab116027)
Even lanes – **triceps brachii muscle of sedentary rats**
Odd lanes – **triceps brachii muscle of voluntary exercise-trained rats**
→ - membrane was cut into three parts:
between 75 and 48 kDa (top) for NOX4 (67 kDa);
between 48 and 28 kDa (middle) for SOD3 (35 – 40 kDa);
below 28 kDa (bottom) for SOD1 (18 kDa).

Top fragment of membrane 8 stained against NOX4

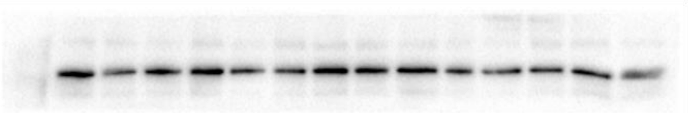

Middle fragment of membrane 8 stained against SOD3

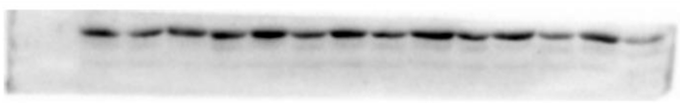

Bottom fragment of membrane 8 stained against SOD1

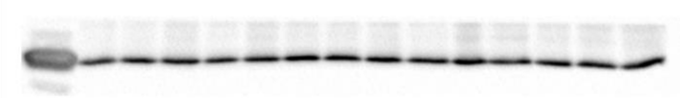

Supplementary FIGURE S5 Ponceau S staining and original images of membrane fragments stained with antibodies against NOX4, SOD3 and SOD1. Membrane 7 – in long head of triceps brachii muscle and diaphragm muscle of sedentary rats (n=7); membrane 8 – in long head of triceps brachii muscle of sedentary (n=7) and voluntary exercise-trained (n=7) rats. Fragments of the membrane (at the level of the NOX4, SOD3, SOD1) were cut out to reduce the quantity of antibodies used.

Membrane 9 after Ponceau S staining

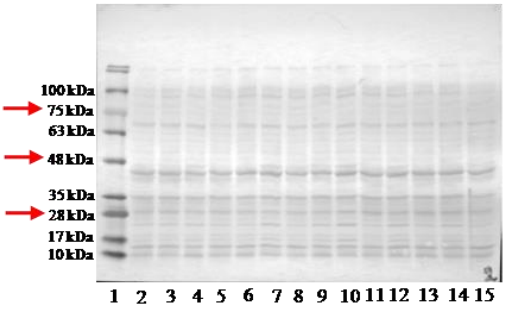

Lane 1: protein ladder (ab116027)
Odd lanes – **diaphragm muscle of sedentary rats**
Even lanes – **diaphragm muscle of voluntary exercise-trained rats**
→ - membrane was cut into three parts:
between 75 and 48 kDa (top) for NOX4 (67 kDa);
between 48 and 28 kDa (middle) for SOD3 (35 – 40 kDa);
below 28 kDa (bottom) for SOD1 (18 kDa).

Top fragment of membrane 9 stained against NOX4

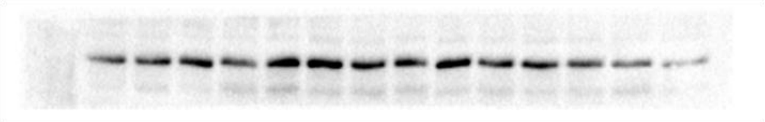

Middle fragment of membrane 9 stained against SOD3

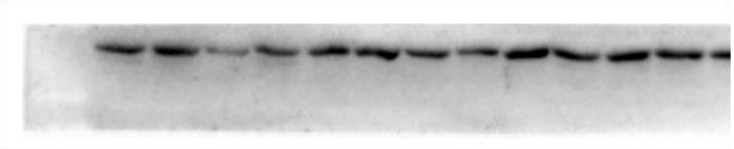

Bottom fragment of membrane 9 stained against SOD1

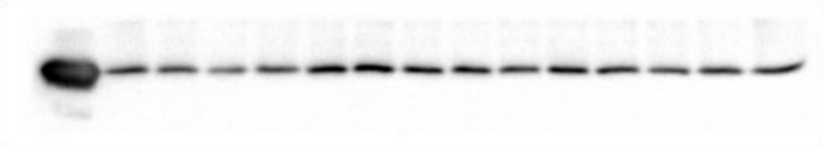

Supplementary FIGURE S6 Ponceau S staining and original images of membrane fragments 9 stained with antibodies against NOX4, SOD3 and SOD1. Membrane 9 – in diaphragm muscle of sedentary (n=7) and voluntary exercise-trained (n=7) rats. Fragments of the membrane (at the level of the NOX4, SOD3, SOD1) were cut out to reduce the quantity of antibodies used.

Membrane 10 after Ponceau S staining

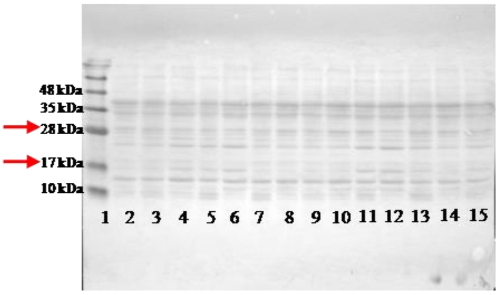

Lane 1: protein ladder (ab116027)
Even lanes: **triceps brachii muscle of sedentary rats**
Odd lanes: **diaphragm muscle of sedentary rats**
→ - membrane was cut between 28 kDa (top) and 17 kDa (bottom) for further visualization of SOD2 (25 kDa).

The fragment of membrane 10 stained against SOD2

Membrane 11 after Ponceau S staining

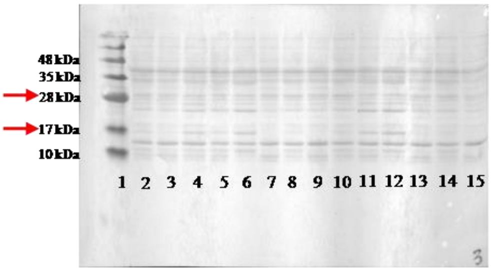

Lane 1: protein ladder (ab116027)
Even lanes: **triceps brachii muscle of sedentary rats**
Odd lanes: **triceps brachii muscle of voluntary exercise-trained rats**
→ - membrane was cut between 28 kDa (top) and 17 kDa (low) for further visualization of SOD2 (25 kDa).

The fragment of membrane 11 stained against SOD2

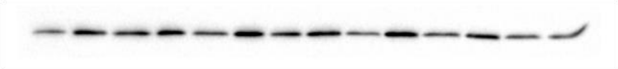

Membrane 12 after Ponceau S staining

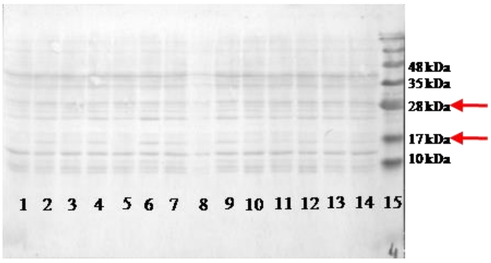

Lane 15: protein ladder (ab116027)
Odd lanes: **diaphragm muscle of sedentary rats**
Even lanes: **diaphragm muscle of voluntary exercise-trained rats**
→ - membrane was cut between 28 kDa (top) and 17 kDa (low) for further visualization of SOD2 (25 kDa).

The fragment of membrane 12 stained against SOD2

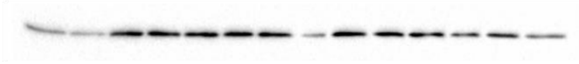

Supplementary FIGURE S7 Ponceau S staining and unprocessed images of membrane fragments stained with antibodies against SOD2. Membrane 10 – in long head of triceps brachii muscle and diaphragm muscle of sedentary rats (n=7); membrane 11 – in long head of triceps brachii muscle of sedentary (n=7) and voluntary exercise-trained (n=7) rats; membrane 12 – in diaphragm muscle of sedentary (n=7) and voluntary exercise-trained (n=7) rats. Fragment (at the level of the SOD2) was cut from the membrane to reduce the quantity of antibodies used.
